# High-Color-Temperature Lighting Is Associated With Activation of MAPK/ERK–nNOS Signaling and MMP-2–Related Pathways in Ocular Tissues

**DOI:** 10.1167/iovs.67.6.46

**Published:** 2026-06-24

**Authors:** Yun-Wei Chiang, Jia-Yuan Chang, Kun-Lin Yeh, Rui-Yu Lin, Shang-Min Yeh

**Affiliations:** 1 Department of Optometry, Central Taiwan University of Science and Technology, Taichung, Taiwan; 2 PhD Program of Electrical and Communications Engineering, Feng Chia University, Taichung, Taiwan; 3 Department of Optometry, University of Kang Ning, Taipei, Taiwan; 4 Department of Veterinary Medicine, College of Veterinary Medicine, National Chung Hsing University, Taichung, Taiwan

**Keywords:** color temperature, ocular remodeling, MAPK/ERK signaling, nitric oxide synthase, myopia

## Abstract

**Purpose:**

To investigate whether exposure to artificial lighting with different correlated color temperatures (CCTs) affects ocular structure and myopia-related molecular signaling pathways before axial elongation in a murine model.

**Methods:**

C57BL/6 mice were exposed to standard lighting (control) or artificial lighting at 3000, 4000, or 6000 K under a 12-hour light/12-hour dark cycle for 21 days. Spectral power distribution and illuminance were recorded. Histological analyses were performed to assess corneal epithelial thickness, retinal outer and inner nuclear layers (ONL and INL), and sclera thickness. Western blotting was used to evaluate phosphorylated extracellular signal-regulated kinase 1/2 (p-ERK1/2), neuronal nitric oxide synthase (nNOS), matrix metalloproteinase-2 (MMP-2), TNF-α, and IL-6. Immunohistochemistry was performed to assess ionized calcium-binding adapter molecule 1 (Iba-1) immunoreactivity in retinal tissues.

**Results:**

Exposure to different CCTs did not affect systemic growth, axial length, or ocular structural integrity. Axial length was comparable across groups (*P* = 0.431), and no significant differences were observed in sclera thickness, ONL, INL, or corneal epithelial thickness. In contrast, molecular analyses revealed CCT-dependent alterations. Exposure to 6000 K lighting increased p-ERK1/2, nNOS, MMP-2, TNF-α, and IL-6 expression and elevated Iba-1 immunoreactivity in retinal tissues.

**Conclusions:**

High-CCT lighting is associated with alterations in myopia-related molecular signaling in the absence of detectable structural or axial changes. These findings highlight early, pre-structural molecular responses to spectral light environments and suggest that CCT influences myopia-relevant pathways before overt ocular remodeling occurs.

Myopia is a growing global public health concern, particularly in East Asia.^1^^,^^2^ High myopia substantially increases the risk of irreversible visual complications, including retinal detachment and myopic maculopathy.^3^ Accordingly, identifying modifiable environmental risk factors has become a major research priority.

Myopia development is multifactorial, involving complex interactions among genetic susceptibility, near-work activities, outdoor exposure, and light-related environmental factors.^3^^,^^4^ Among these, increased time spent outdoors has been consistently associated with a reduced risk of myopia onset and progression.^5^^–^^7^ The protective effects of outdoor activity have been attributed to higher ambient light intensity, the broader spectral profile of natural daylight, and activation of retinal biochemical signaling pathways involving neurotransmitters such as dopamine, which influence downstream scleral remodeling processes, including the regulation of matrix metalloproteinase (MMP) expression.^8^ Importantly, experimental evidence indicates that light exposure modulates retinal and scleral signaling cascades involved in ocular growth regulation.^9^^,^^10^

Prior investigations into light-related mechanisms of myopia have largely examined the effects of specific wavelengths under controlled experimental conditions. Short-wavelength light suppresses axial elongation in certain animal models, whereas longer-wavelength light promotes ocular growth in others.^11^^,^^12^ However, these effects vary across species, developmental stages, and experimental paradigms, indicating that wavelength-specific responses are context dependent. Moreover, exposure to isolated narrow-band wavelengths does not accurately reflect the spectral characteristics of real-world lighting environments.

Correlated color temperature (CCT), expressed in Kelvin (K), provides an integrated descriptor of the spectral composition of polychromatic light sources and represents a practical, environmentally relevant parameter for indoor lighting. Unlike single-wavelength exposure, CCT reflects the combined spectral output of commonly used artificial light sources and is readily adjustable in residential, educational, and occupational settings. Despite growing interest in the role of indoor lighting in myopia development, it remains unclear whether differences in CCT modulate early retinal and ocular molecular signaling pathways associated with myopia, particularly before measurable axial elongation occurs

Accordingly, this study investigated the effects of exposure to different CCT lighting conditions on ocular structure and myopia-related molecular signaling in a murine model. We focused on candidate pathways implicated in early myopia-associated signaling and extracellular matrix regulation, including MAPK/ERK phosphorylation and nitric oxide-related signaling (nNOS), as well as the downstream extracellular matrix modulator matrix metalloproteinase-2 (MMP-2).^13^^,^^14^ In parallel, we assessed inflammatory mediators (IL-6 and TNF-α), given evidence linking inflammatory signaling to myopia-associated ocular responses and its potential interaction with remodeling-related pathways.^15^^,^^16^ By prioritizing these molecular endpoints in the absence of overt structural changes, this study addresses an important knowledge gap regarding early, pre-structural responses to spectral light environments and provides mechanistic insight into how indoor lighting characteristics may influence myopia-related biological processes.

## Methods

### Animal Model and Experimental Design

Four-week-old C57BL/6 mice were randomly assigned to four groups (*n* = 4 per group): a control group maintained under standard laboratory lighting conditions and three experimental groups exposed to artificial lighting with CCTs of 3000, 4000, or 6000 K. This developmental stage represents an active phase of postnatal ocular growth and is used in experimental myopia research to evaluate early molecular responses to environmental modulation before overt axial elongation occurs.^17^^,^^18^ Mice were housed under a 12-hour light/12-hour dark cycle for 21 consecutive days, with food and water provided as desired. The 21-day light exposure period was selected based on prior experimental studies indicating that early ocular and molecular responses to visual environment manipulation can be detected after short-term exposure periods in animal models.^11^^,^^17^

### Lighting Setup and Characterization

**Figure 1.**
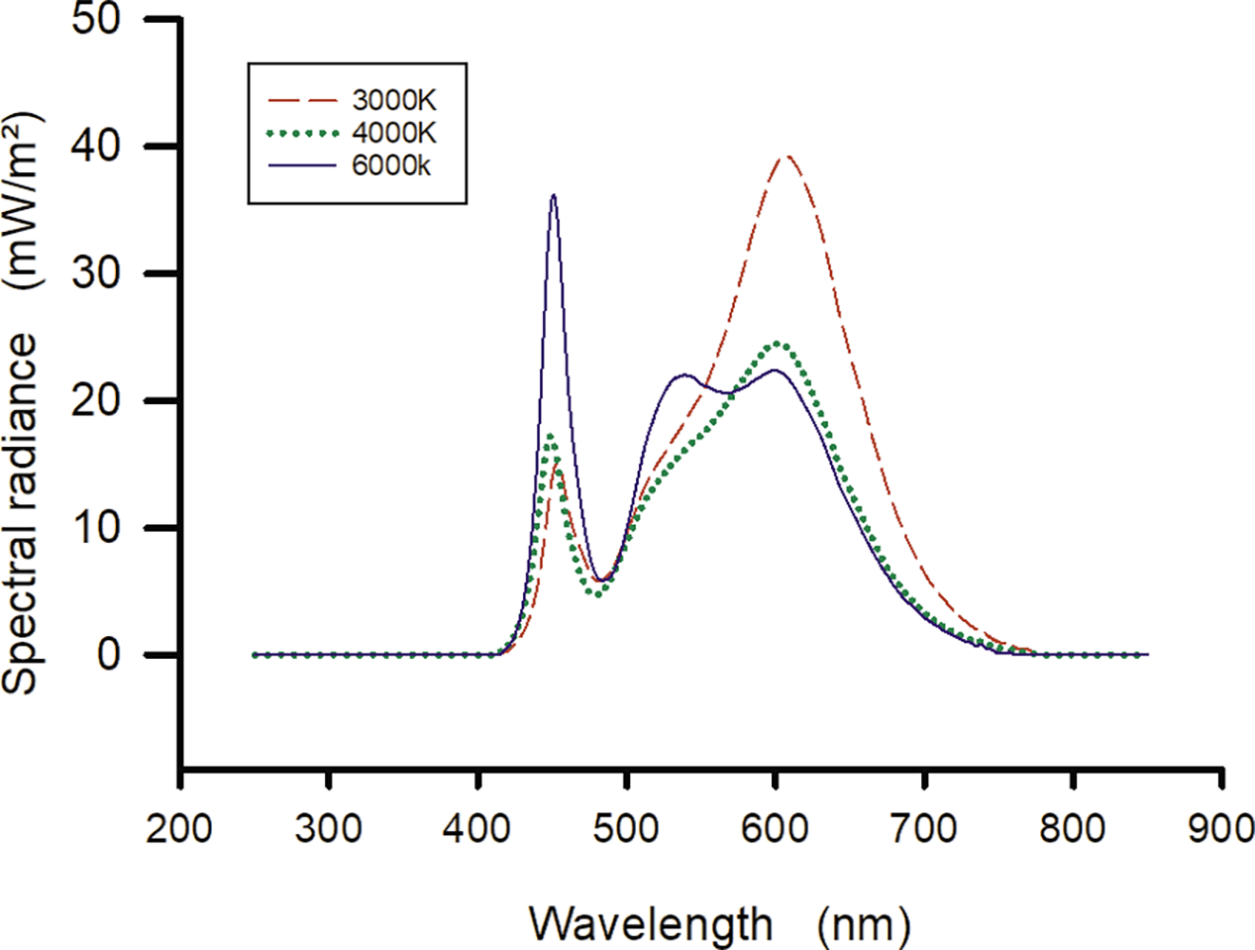
Spectral radiance distributions of the three lighting conditions. *Dashed line*: the 3000 K light source; *dotted line*: the 4000 K light source; *solid line*: the 6000 K light source.

To characterize and standardize the lighting environment, the spectral power distribution of each light source was measured using a spectroradiometer (Optimum SRI-2500TR; Optimum Optoelectronics Corp., Chubei, Taiwan), and photopic illuminance was measured at the cage level using a calibrated digital lux meter (TES 1330A; TES Electrical Electronic Corp., Taipei, Taiwan). Measurements were obtained at multiple locations within each cage and averaged to account for spatial variation. Lighting fixtures were mounted directly above the cages at a fixed distance of 25 cm from the cage floor to ensure uniform illumination across all experimental conditions. All lighting systems were calibrated before the experiment and monitored periodically throughout the 21-day exposure period to maintain stable spectral output and irradiance. Spectral power distributions for each CCT condition are presented in Figure 1. Illuminance at the cage floor level was approximately 1698.5 ± 37.9 lx for the 3000 K condition, 1610.5 ± 48.2 lx for the 4000 K condition, and 1646.8 ± 42.3 lx for the 6000 K condition. Statistical analysis confirmed no significant differences in photopic illuminance among the three CCT conditions (*P* > 0.05).

### Axial Length Measurement

Axial length (AL) was measured on enucleated eyes using an electronic digital caliper (Krisbow), following the procedure described by Wahyuningsih et al.^19^ Baseline AL measurements (Day 0) were obtained from an independent cohort of four-week-old mice before light exposure, and endpoint measurements were obtained at Day 21 after completion of the experimental protocol. After euthanasia, eyes were carefully enucleated and rinsed with phosphate-buffered saline solution. For each eye, the caliper jaws were positioned at the corneal apex and posterior pole of the globe. Care was taken to avoid compressing or deforming the eyeball during measurement (Supplementary Figure S1). Each eye was measured three times independently by two trained examiners, and the mean value was used for analysis. Examiners were masked to the experimental group allocation. Enucleated eyes were coded, and group information was not disclosed during measurement to minimize bias. The caliper had a measurement resolution of 0.01 mm, and the same device was used throughout the study to ensure consistency. All measurements were completed within 10 min of enucleation to minimize tissue dehydration-related changes.

### Histological Analysis

After euthanasia, eyes were enucleated, fixed, and embedded in paraffin. Tissue sections were processed and stained with hematoxylin and eosin (H&E) using standard protocols, including deparaffinization, graded ethanol rehydration, hematoxylin staining, rinsing with tap water, eosin counterstaining, dehydration, xylene clearing, and mounting. H&E-stained sections were digitized using a whole-slide imaging system and analyzed using CaseViewer software (version 2.4; 3DHISTECH, Budapest, Hungary). Corneal epithelial thickness was measured at multiple predefined locations at anatomically comparable positions within each section. For scleral thickness, outer nuclear layer (ONL), and inner nuclear layer (INL) analysis, measurements were performed at anatomically comparable locations within the posterior retinal and scleral regions using consistent regional landmarks, and the mean value from multiple sites per section was used for statistical analysis.

### Immunohistochemistry

Immunohistochemical staining for ionized calcium-binding adaptor molecule 1 (Iba-1) was performed on paraffin-embedded ocular sections. After deparaffinization and rehydration, heat-induced antigen retrieval was conducted in citrate buffer (pH 6.0). Endogenous peroxidase activity was blocked before incubation with primary anti-Iba-1 antibody (sc-32725, 1:50 dilution; Santa Cruz Biotechnology, Dallas, TX, USA) for 1.5 hour at room temperature. Signal detection was conducted using a horseradish peroxidase –polymer system with 3,3′-diaminobenzidine (DAB) visualization, followed by hematoxylin counterstaining. Negative controls were processed without primary antibody under identical staining conditions.

Whole-slide images were digitized and analyzed using CaseViewer software (version 2.4; 3DHISTECH). Quantitative analysis of Iba-1 immunoreactivity was performed using Fiji (ImageJ; National Institutes of Health, Bethesda, MD, USA). Color deconvolution (H-DAB) was applied to isolate DAB staining, and a uniform threshold was applied across all sections. The percentage of Iba-1-positive area was calculated within predefined regions of interest (ROIs) in the posterior retinal region at anatomically comparable locations across sections. Three non-overlapping ROIs were analyzed per section, and the mean value per eye was used for statistical analysis. Image selection and quantification were performed in a masked manner. Because Iba-1 labels both microglial somata and cellular processes, the percentage of Iba-1 positive area was used to quantify overall immunoreactivity in retinal sections, consistent with previous studies of retinal microglial activation.^20^^,^^21^

### Protein Extraction and Western Blot Analysis

Ocular tissues were homogenized in T-PER buffer containing protease and phosphodiesterase inhibitors. Lysates were centrifuged, and the supernatant was collected for protein quantification using a bicinchoninic acid assay. Equal amounts of protein (25 µg per lane) were separated on 4%–12% Bis-Tris sodium dodecyl sulfate–polyacrylamide gel electrophoresis gels and transferred onto polyvinylidene difluoride membranes.

Membranes were blocked with 5% skim milk in Tris-buffered saline containing 0.1% Tween 20 (TBS-T) and incubated overnight at 4°C with primary antibodies against MMP-2, nNOS, IL-6, phosphorylated extracellular signal–regulated kinase 1/2 (p-ERK1/2), and TNF-α. After incubation with horseradish peroxidase–conjugated secondary antibodies, protein bands were visualized using enhanced chemiluminescence and captured with a digital imaging system. A β-actin was used as the internal loading control.

Densitometric analysis of immunoreactive bands was performed using iBright Analysis Software (Thermo Fisher Scientific, Waltham, MA, USA). Band intensities were normalized to the corresponding loading control for each sample. All samples for each target protein and their corresponding β-actin loading controls were captured under identical exposure settings within each experiment to ensure consistent quantification.

### Statistical Analysis

Data are presented as mean ± SEM. Statistical analyses were conducted using IBM SPSS Statistics (version 22; IBM Corp., Armonk, NY, USA). Group differences were assessed using one-way analysis of variance. When a significant main effect was observed, Bonferroni post hoc tests were applied for pairwise comparisons. A *P*-value < 0.05 was considered statistically significant.

## Results

### Effects of Color Temperature on Animal Growth and Ocular Tissue Health

All mice were four weeks old at treatment initiation. Baseline body weights did not differ significantly among groups (Control: 17.77 ± 0.18*g*; 3000 K: 17.97 ± 0.50*g*; 4000 K: 18.34 ± 0.11*g*; 6000 K: 16.77 ± 0.12*g*; *P* = 0.100). After three weeks of intervention, all groups demonstrated normal growth progression, with no statistically significant differences in final body weight (Control: 23.39 ± 0.94*g*; 3000 K: 22.46 ± 1.11*g*; 4000 K: 23.21 ± 0.46*g*; 6000 K: 21.40 ± 1.44*g*; *P* = 0.544) (Supplementary Fig. S2). These findings indicate that CCT exposure did not affect systemic growth or body size, suggesting that the lighting intervention did not induce physiological stress.

**Figure 2.**
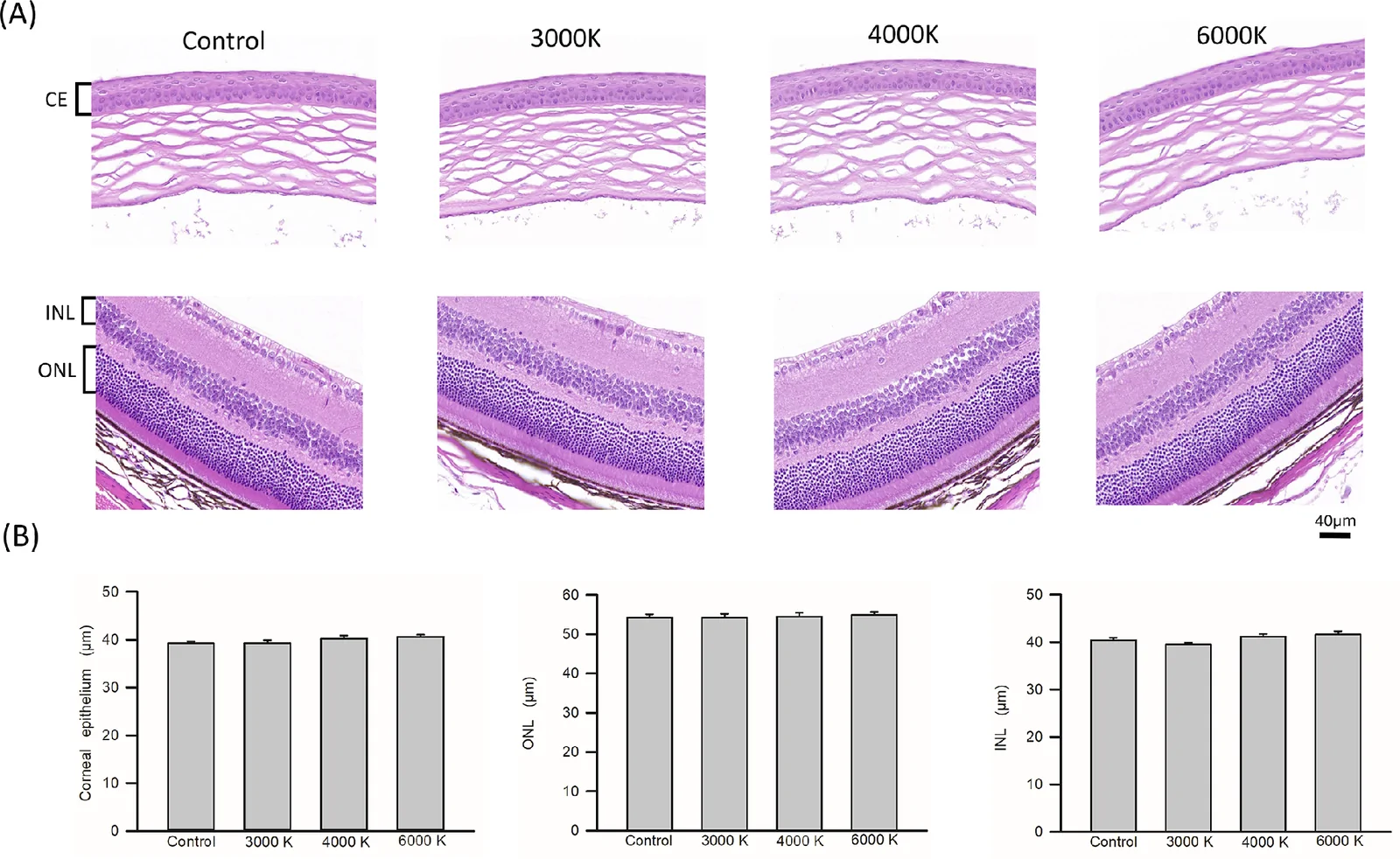
Effects of different CCT lighting conditions on corneal and retinal layer thickness. **(A)** Representative H&E-stained images of the corneal epithelium (CE) (*top row*) and retinal layers including the ONL and INL (*bottom row*) from the control, 3000 K, 4000 K, and 6000 K groups after 21 days of light exposure. *Scale bar*: 40 µm. **(B)** Quantification of corneal epithelial, outer nuclear layer thickness, and inner nuclear layer thickness; no significant differences were observed among groups. Data are presented as mean ± SE (*n* = 4 mice per group).

Histological analysis revealed no significant differences in corneal epithelial thickness, ONL, or INL among the groups. Thus exposure to varying CCT conditions did not compromise corneal or retinal health (Fig. 2).

### Effects of Color Temperature on Axial Length

After 21 days, axial length did not differ significantly among the experimental groups (control: 3.20 ± 0.04 mm; 3000 K: 3.23 ± 0.02 mm; 4000 K: 3.27 ± 0.03 mm; 6000 K: 3.25 ± 0.02 mm; *P* = 0.431) (Table). Therefore CCT did not significantly influence axial elongation during the experimental period.

**Table.** Baseline and Endpoint Axial Length Measurements After 21 Days of Color Temperature Exposure

	Baseline (Day 0)	Control	3000K	4000K	6000K
AL (mm)	3.11 ± 0.01	3.20 ± 0.04^*^	3.23 ± 0.02^*^	3.27 ± 0.03^*^	3.25 ± 0.02^*^

* Significantly different from baseline (Day 0), *P* < 0.05

Baseline AL measured at Day 0 in 4-week-old mice was 3.11 ± 0.01 mm. At Day 21, AL was significantly greater than baseline in all groups (*P* < 0.05), reflecting normal age-related ocular growth. However, no significant differences were observed among CCT groups.

### Effects of Color Temperature on Scleral Thickness

**Figure 3.**
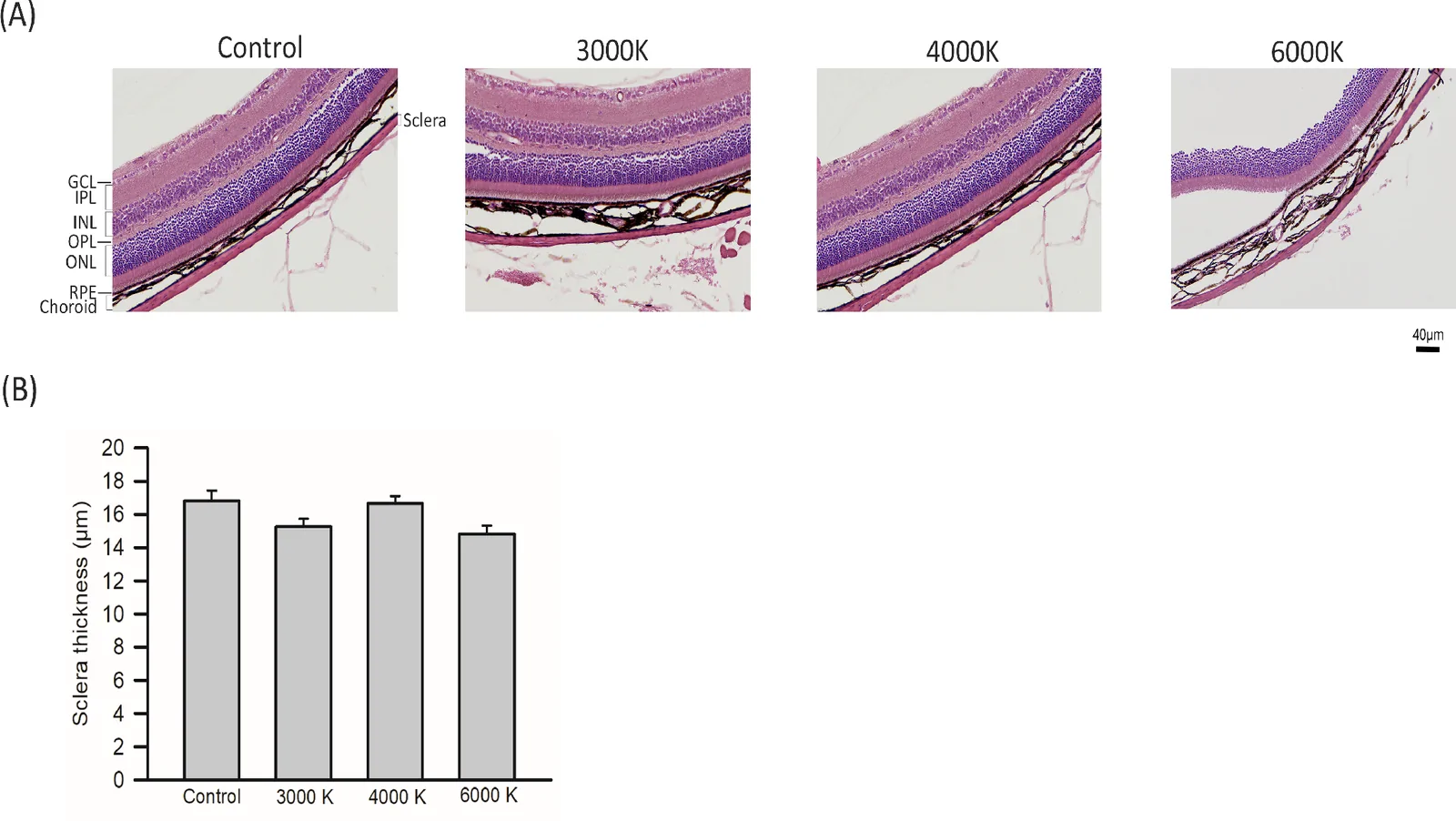
Effects of CCT exposure on sclera thickness. **(A)** Representative H&E-stained sections of the posterior eye wall from the control, 3000 K, 4000 K, and 6000 K groups after 21 days of CCT exposure. Retinal layers including the ganglion cell layer (GCL), inner plexiform layer (IPL), INL, outer plexiform layer (OPL), ONL, retinal pigment epithelium (RPE), choroid and sclera are indicated. *Scale bar*: 40 µm. **(B)** Quantitative comparison of sclera thickness showing no statistically significant differences. *Double arrows* indicate sclera. Data are presented as mean ± SE (*n* = 4 mice per group).

Quantitative analysis of H&E-stained sections revealed no statistically significant differences in sclera thickness among the four groups after 21 days of exposure. The mean sclera thicknesses were 16.81 ± 0.63 µm (control), 15.26 ± 0.47 µm (3000 K), 16.66 ± 0.44 µm (4000 K), and 14.83 ± 0.50 µm (6000 K) (*P* = 0.202) (Fig. 3).

### Effects of Color Temperature on Inflammatory Factors TNF-α and IL-6

**Figure 4.**
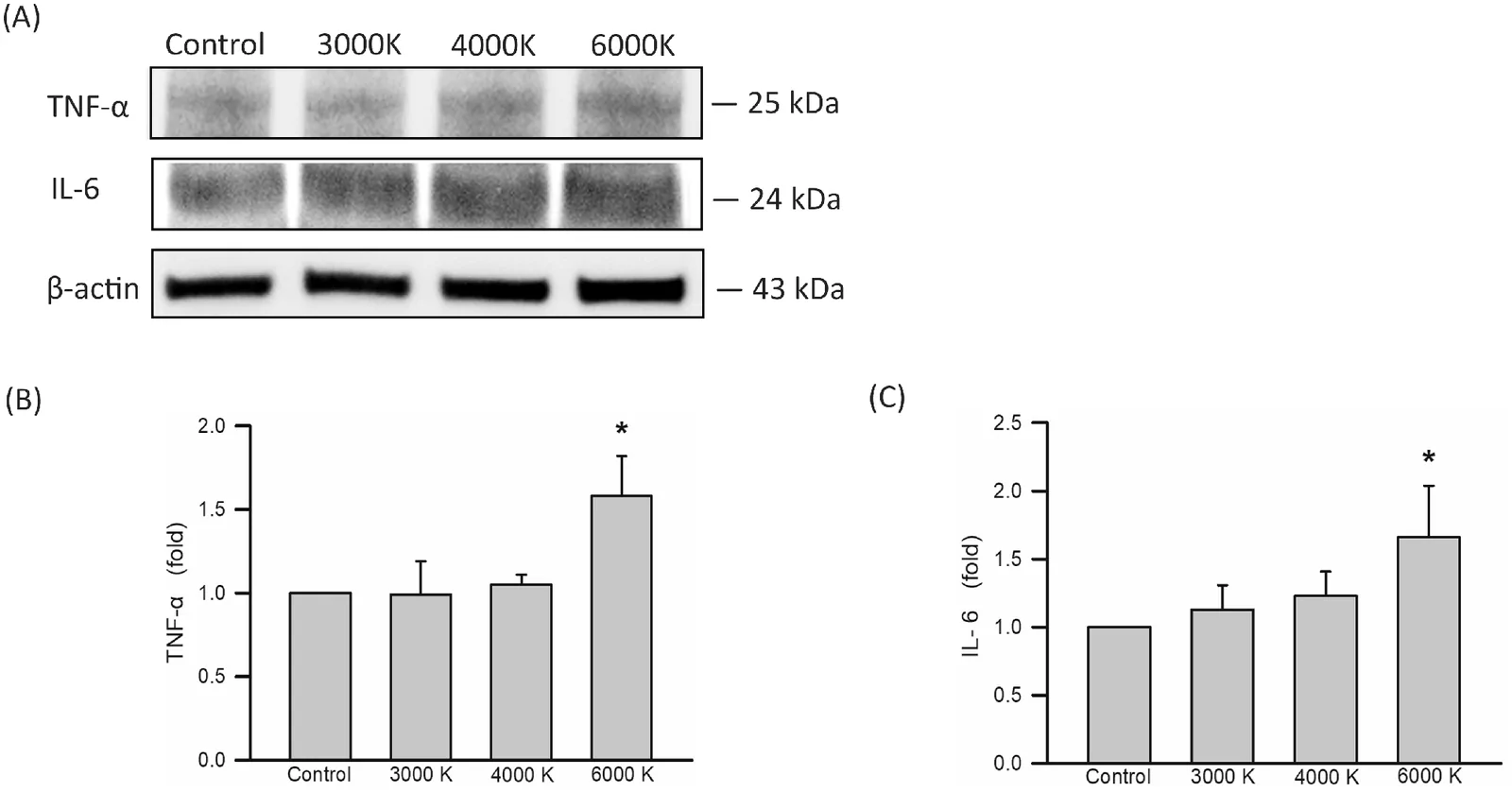
Effects of CCT exposure on inflammatory marker expression in ocular tissues. **(A)** Western blot images of TNF-α and IL-6 expression in the control, 3000 K, 4000 K, and 6000 K groups after 21 days of light exposure. **(B, C)** Quantification of TNF-α and IL-6 expressions showing significantly increased levels in the 6000 K group compared with the control group (*P* = 0.042 and *P* = 0.043, respectively). Data are presented as mean ± SE (*n* = 4 mice per group). *P < 0.05 versus the control group.

Western blot analysis demonstrated that high-CCT exposure modulated inflammatory protein expression in ocular tissues. TNF-α levels were significantly elevated in the 6000 K groups compared with the control group (*P* = 0.042). Similarly, IL-6 expression was significantly increased in the 6000 K group (*P* = 0.043), whereas the 3000 and 4000 K groups did not differ significantly from the control (Fig. 4).

### Effects of Color Temperature on Iba-1 Immunoreactivity

**Figure 5.**
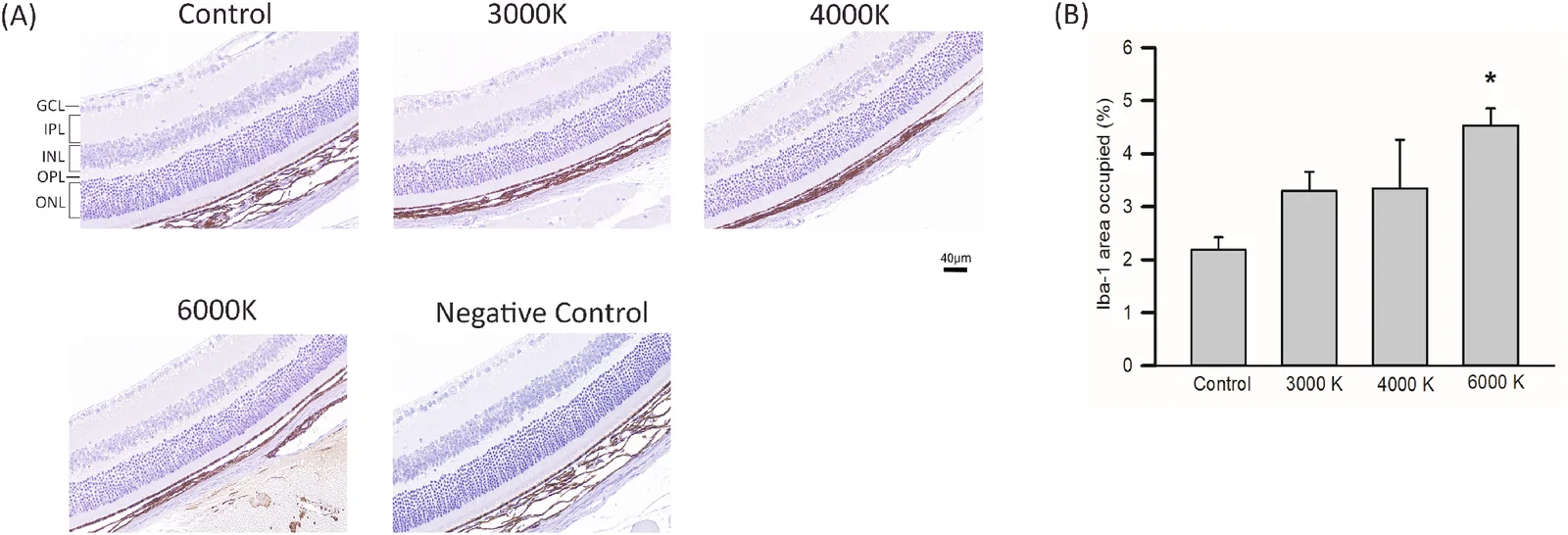
Effects of CCT on Iba-1 immunoreactivity in retinal tissues. **(A)** Representative immunohistochemical staining of Iba-1 in retinal sections from control, 3000 K, 4000 K, and 6000 K groups after 21 days of exposure. Iba-1–positive cells are visualized by DAB staining (*brown*), with hematoxylin counterstaining (*blue*). A negative control section processed without primary antibody is shown for comparison. Retinal layers including the ganglion cell layer (GCL), inner plexiform layer (IPL), INL, outer plexiform layer (OPL), and ONL are indicated. *Scale bar*: 40 µm. **(B)** Quantification of Iba-1 immunoreactivity expressed as percentage of Iba-1-positive area within predefined posterior retinal regions of interest. The 6000 K group exhibited a significant increase in Iba-1-positive area compared with the control (*P* < 0.05). Data are presented as mean ± SE (*n* = 4 mice per group). *P < 0.05 versus the control group.

Immunohistochemical analysis demonstrated that high-CCT exposure was associated with increased Iba-1 immunoreactivity in the posterior retinal region. The percentage of Iba-1-positive area was significantly increased in the 6000 K group compared with the control group (*P* = 0.009), consistent with an expansion of Iba-1–positive immunoreactive area in the posterior retinal region. In contrast, the 3000 and 4000 K groups did not differ significantly from the control (Fig. 5).

### Effects of Color Temperature on Extracellular Matrix Remodeling Mediated by MMP-2

**Figure 6.**
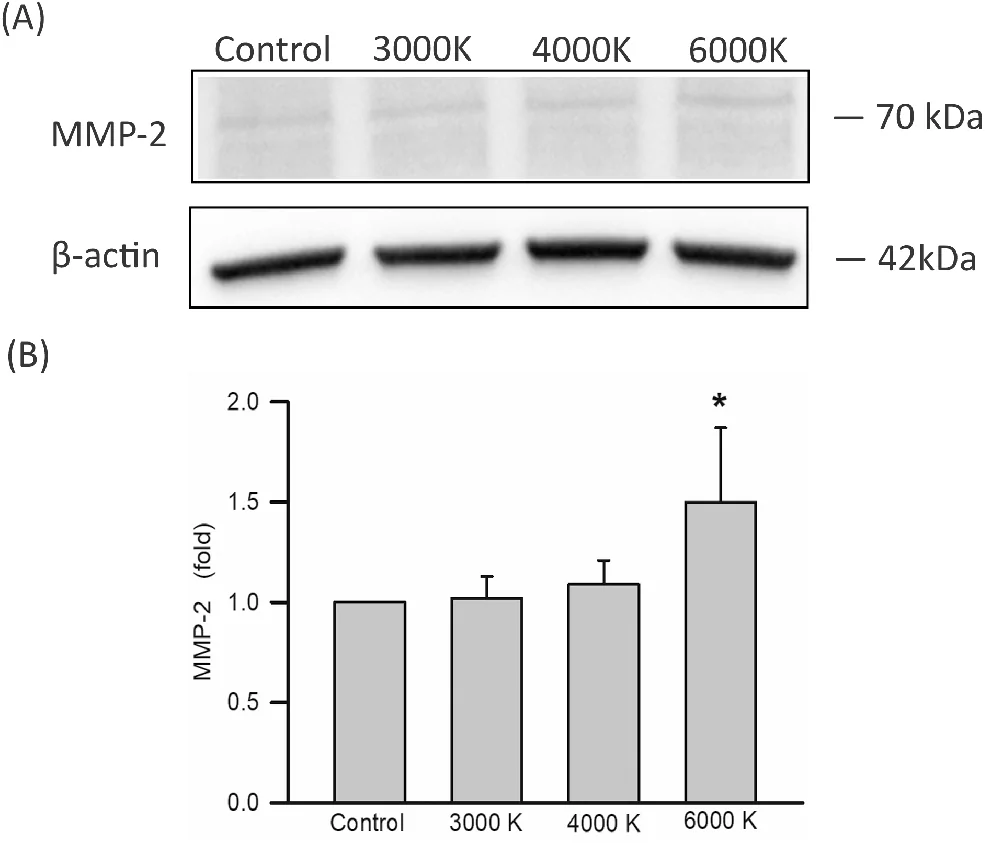
Effects of CCT exposure on MMP-2 expressions in ocular tissues. **(A)** Western blot images showing MMP-2 protein expression in the control, 3000 K, 4000 K, and 6000 K groups after 21 days of light exposure. **(B)** Quantification of MMP-2 expression, indicating a significant increase in the 6000 K groups compared with the control group (*P* = 0.011). Data are presented as mean ± SE (*n* = 4 mice per group). *P < 0.05 versus the control group.

MMP-2 expression was significantly increased in the 6000 K groups compared with the control group (*P* = 0.011; Fig. 6). Elevated MMP-2 expression was observed in association with high-CCT exposure, a molecular change that has been reported in the context of extracellular matrix remodeling processes involved in scleral structural alterations during myopia development.

### Effects of Color Temperature on Nitric Oxide Signaling and MAPK/ERK Pathway

**Figure 7.**
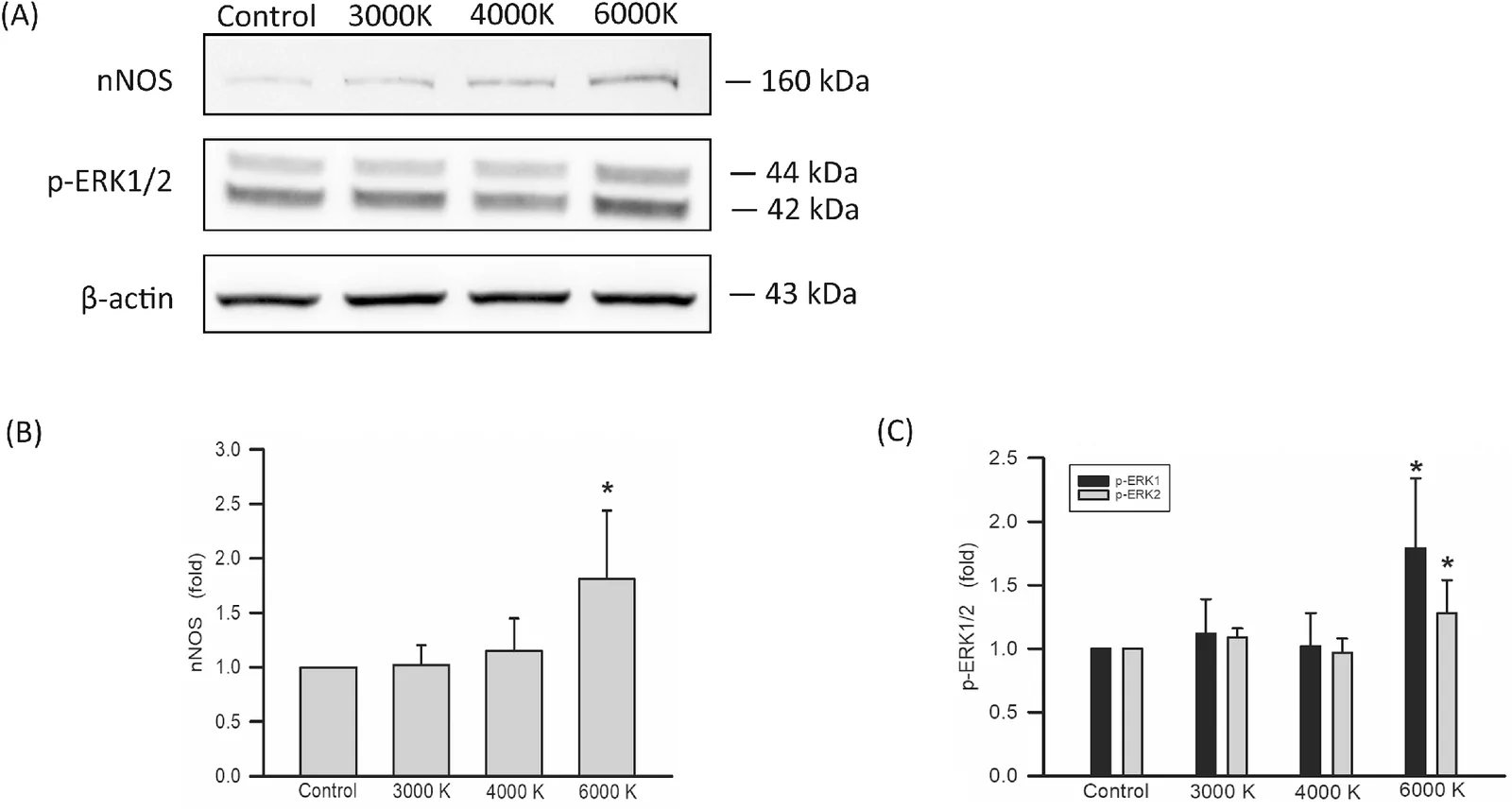
Effects of CCT exposure on nNOS signaling and MAPK/ERK pathway activation in ocular tissues. **(A)** Western blot images illustrating nNOS and p-ERK1/2 expression in the control, 3000 K, 4000 K, and 6000 K groups after 21 days of light exposure. (**B**, **C**) Quantification of nNOS and p-ERK1/2 expression, showing a significant increase in the 6000 K group compared with the control group (*P* < 0.05). Data are presented as mean ± SE (*n* = 4 mice per group). *P < 0.05 versus the control group.

Only the 6000 K group exhibited a significant increase in nNOS expression (*P* = 0.037) compared with the control, accompanied by elevated p-ERK1/2 levels (Fig. 7). These molecular changes were observed in association with high-CCT exposure, reflecting concurrent modulation of nitric oxide–related signaling and MAPK/ERK pathway activity in ocular tissues.

## Discussion

This study investigated the effects of exposure to different CCT lighting conditions (3000, 4000, and 6000 K) on ocular tissues in mice, with emphasis on molecular signaling pathways implicated in myopia-related ocular growth. Across all experimental groups, no significant differences were observed in systemic growth parameters or major ocular structural indices, including AL, retinal layer thicknesses, scleral thickness, and corneal epithelial thickness, after the 21-day exposure period. These findings indicate that short-term variation in CCT did not induce detectable morphological or refractive alterations in the murine eye.

The absence of overt structural changes does not exclude early biological responses to visual environments. Experimental evidence has demonstrated that molecular and cellular alterations relevant to myopia can precede detectable morphological remodeling. In avian models, chromatic manipulation of ambient light induces progressive refractive changes within approximately two to three weeks, accompanied by alterations in ocular growth trajectories.^11^ Similarly, in murine models, form-deprivation paradigms initiated at approximately four weeks of age trigger early myopia-associated biological responses, including scleral immune cell polarization and extracellular matrix-related signaling changes, within two weeks.^17^ Together, these findings support the interpretation that short-term visual or spectral manipulation can initiate early molecular and cellular responses associated with myopia development, even in the absence of detectable axial elongation or structural remodeling. The 21-day exposure period in this study was sufficient to detect early molecular responses, although longer exposure may be required to evaluate structural consequences.

Outdoor light exposure is associated with a reduced risk of myopia onset and progression, with epidemiological studies consistently reporting lower myopia prevalence among children who spend more time outdoors.^5^^,^^6^ This association has been hypothesized to relate to higher light intensities and broader spectral characteristics of natural daylight.^22^ Animal studies further suggest that warmer, low-color-temperature artificial lighting (approximately 2700–3000 K) is associated with reduced axial elongation compared with higher CCT ranges (approximately 4000–5000 K), particularly under prolonged exposure.^23^ In addition, reviews of CCT-related studies indicate that high-CCT artificial lighting environments may be associated with greater myopia progression in humans. However, these reviews also emphasize that the associations are subject to potential confounding factors and that the number of available and methodologically comparable studies remains limited.^24^ Within this broader context, the present findings provide molecular-level evidence supporting a role for spectral light environments in ocular growth regulation.

Multiple signaling pathways have been implicated in experimental myopia and ocular tissue remodeling. Nitric oxide-related signaling, MAPK/ERK activation, inflammatory mediators, and extracellular matrix regulators such as MMP-2 interact within a broader interconnected regulatory network. Previous studies have reported increased nNOS activity in form-deprivation myopia models, accompanied by alterations in nitric oxide-related signaling pathways, including the guanylate cyclase–cyclic guanosine monophosphate cascade.^25^^,^^26^ PI3K/AKT/ERK signaling has also been implicated in experimental myopia-associated fibrotic remodeling, with MEK–ERK activation leading to upregulation of downstream effectors such as nNOS and MMP-2, alongside inflammatory and oxidative stress-related pathways.^14^^,^^27^ The ERK1/2–MMP-2 axis has been proposed as a contributor to posterior scleral remodeling in form-deprivation myopia models, linking ERK activation to extracellular matrix degradation.^28^ In this context, the observed CCT-dependent modulation of MAPK/ERK–nNOS signaling, MMP-2 expression, and inflammatory mediators in this study is best interpreted as early molecular responses to the spectral light environment. Future studies incorporating pharmacologic inhibition or genetic manipulation of key signaling components (e.g., MAPK/ERK or nNOS pathways) are needed to determine whether these molecular responses causally mediate CCT-related ocular changes. In addition, longer-term and longitudinal CCT exposure studies will be necessary to assess whether these early molecular alterations translate into measurable changes in ocular growth and development.

Inflammatory and oxidative stress pathways have been implicated in myopia-associated ocular alterations and extracellular matrix remodeling. Elevated IL-6 levels have been reported in the aqueous humor of eyes with longer AL, whereas TNF-α findings are more variable.^29^ Retinal microglia and macrophage-lineage cells are recognized sources of pro-inflammatory cytokines, including IL-6 and TNF-α, and actively participate in retinal immune regulation and tissue remodeling.^30^ Immune cell involvement in scleral remodeling has also been documented in experimental myopia models,^17^ and increased Iba-1 expression has been observed in the brains of highly myopic mice,^31^ suggesting that neuroinflammatory responses accompany myopia-associated changes.

In this study, high-CCT (6000 K) exposure significantly upregulated IL-6 and TNF-α, consistent with an inflammatory response associated with spectral light conditions. Inflammatory cytokines and oxidative stress-related pathways interact with MAPK/ERK and nitric oxide-related cascades, converging on downstream regulators of extracellular matrix turnover such as MMP-2.^14^ TGF-β signaling has also been implicated in myopia-associated ocular remodeling, with links to NF-κB activation and MMP-2 upregulation in experimental myopia models.^32^ Collectively, these findings are consistent with a mechanistic framework in which inflammatory signaling, MAPK/ERK–nNOS activity, and extracellular matrix remodeling may be interconnected. Within this framework, the coordinated upregulation of inflammatory mediators, Iba-1 immunoreactivity, p-ERK1/2, nNOS, and MMP-2 observed under high-CCT exposure in this study is consistent with modulation of early molecular pathways associated with ocular tissue remodeling, preceding detectable structural changes.

This study has a few limitations. First, the relatively short exposure duration limits inference regarding long-term structural outcomes, particularly progressive axial elongation. Second, axial length was measured ex vivo at a single endpoint, precluding baseline and longitudinal assessments and limiting in vivo functional validation. In addition, the relatively small sample size (*n* = 4 per group) may limit the generalizability of the findings. Although molecular alterations were detectable at 21 days, whether prolonged high-CCT exposure would produce measurable structural changes remains uncertain. Previous studies suggest that spectrum-dependent visual signals influence ocular growth over extended developmental periods, with structural outcomes emerging after longer exposure.^33^ Future studies incorporating longer exposure paradigms, non-invasive in vivo biometry, larger cohorts, and multiple time points will be required to determine whether early molecular alterations under high-CCT conditions translate into progressive axial growth.

Despite these limitations, the present findings provide insight into how indoor spectral lighting may influence early molecular pathways relevant to myopia. The coordinated molecular responses observed under high-CCT exposure suggest a potential window during which environmental lighting could modulate myopia-related signaling prior to overt structural remodeling. Further longitudinal and translational studies are required to evaluate the implications of CCT optimization for myopia prevention.

## Supplementary Material

Supplement 1
