# Supplementary material for: High-Color-Temperature Lighting Is Associated With Activation of MAPK/ERK–nNOS Signaling and MMP-2–Related Pathways in Ocular Tissues
Source: Invest Ophthalmol Vis Sci. 2026 Jun 24;67(6):46. doi: 10.1167/iovs.67.6.46 (PMC13313043; doi:10.1167/iovs.67.6.46)
Supplement: Supplement 1 [file iovs-67-6-46_s001.docx]

**
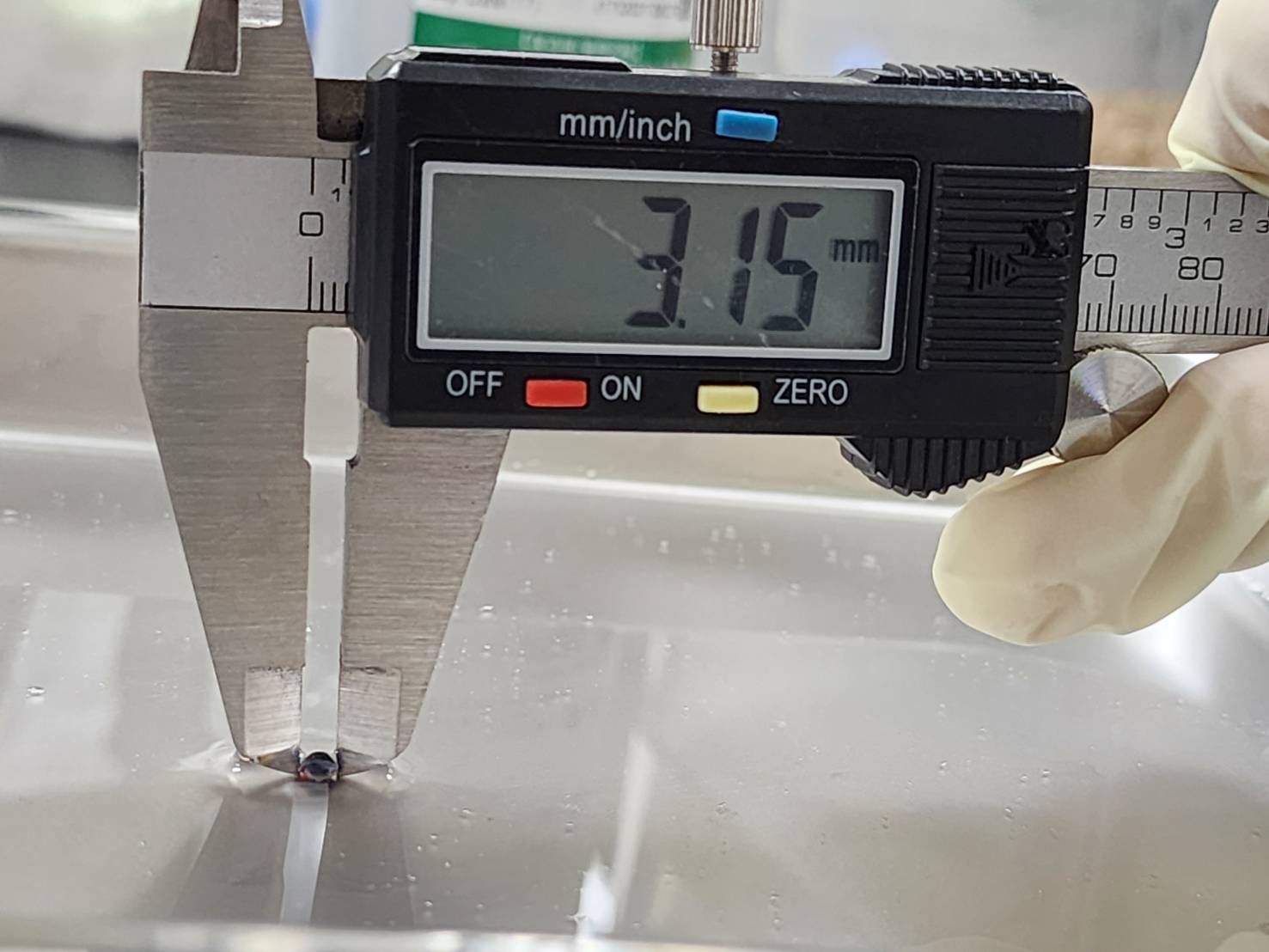
**

**Supplementary Figure S1.** Measurement of mouse axial length using a digital Vernier caliper.

**
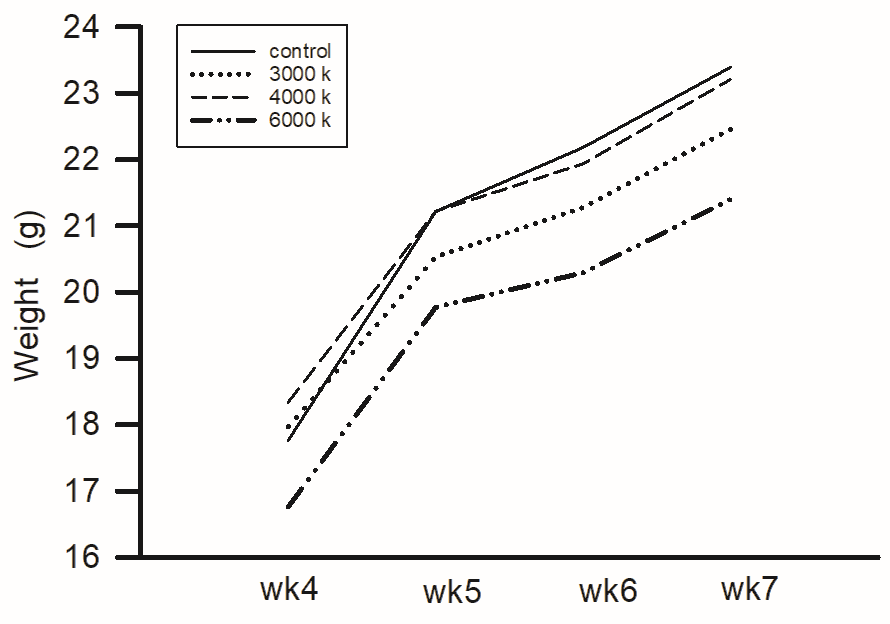
**

**Supplementary Figure S2.** Body weight progression under different correlated color temperature (CCT) lighting conditions. Weekly body weight (g) from week 4 to week 7 is shown for the control, 3000, 4000, and 6000 K groups. All groups exhibited comparable weight gain over the 21-day exposure period, with no significant differences among groups at any time point. Data are presented as mean ± SE (n = 4 mice per group).
